# Indents

**Published:** 1920-09

**Authors:** 


### INDENTS

**☞ Brief Articles of Technical or Scientific Value will receive notice and comment. Contributions invited.**

---

BACTERIA IN TOOTH PULPS Perhaps the most striking announcement of the year is that of Henrici and Hartzell, who have found living bacteria in a large number of living tooth pulps. In an examination of a quantity of extracted teeth they sterilized the outer surfaces of the teeth, cracked them open and tested the pulp for bacteria. In no case could they find bacteria in the pulps of teeth which were fully normal; *i. e.*, unaffected by caries or pyorrhea. But in those teeth which were affected by caries, pyorrhea, or both, 42 to 46 per cent. of the pulps were infected, the most common organism being streptococcus viridans. In regard to their startling claims, they say: "If our observations are correct, we must conclude that in approximately one-half the number of vital teeth invaded by caries or surrounded by pyorrhea the pulp is already infected by streptococci, a conclusion so startling that we felt some hesitation about placing our work on record as this time. Nevertheless we can find no fault in our technic, supported as it is by a series of twenty-two normal controls with uniformly negative results." These findings are indeed startling and they by all means should be checked and corroborated by further investigation of this question. If it should be proven that bacterial invaders frequently gain entrance to the pulps of teeth seriously affected with caries and pyorrhea, it may come to pass that our present conceptions of pulp conservation for such teeth may undergo a radical change. The time may come when we will prefer a well-filled sterile root-canal to one that is filled with an infected pulp. But for the present let us reserve our judgment upon the matter until the facts of the case are fully established.—*Journal N. D. A.*

Is a Child's Life Worth Six Dollars? How much does it cost to prevent deaths from diphtheria? The Ohio *Public Health Journal* for February gives the experience of the city of Salem, Ohio, for twenty years. During the ten years from 1890 to 1899 this town of about ten thousand, the inhabitants had 162 cases of diphtheria with 48 deaths, or 30 per cent. In 1900 the city health authorities began furnishing free antitoxin for all cases of diphtheria. In ten years, from 1900 to 1909, there were 126 cases, with only one death, a case in which antitoxin was not administered until the third day. Had the percentage of fatalities been as high during the second decade as during the first, Salem would have had thirty-seven deaths from diphtheria in that time. The free antitoxin furnished for the ten years cost the city approximately $220, or an average of $6 per life. Any community desiring to know how much it would cost to save a child's life from diphtheria can ascertain this by multiplying the annual number of diphtheria cases by the cost of five thousand units of antitoxin and dividing the result by the number of deaths from diphtheria each year.—Dr. King, in *J. N. D. A.*

---

Are Non-vital Teeth Foreign Bodies? "It has been recently stated on the floor of the Minneapolis District Dental Society by an eminent author that teeth, as soon as the pulps have been removed, immediately become foreign bodies and should be at once extracted.

"A brief review of the embryology and histology of tooth structures and investing tissues of teeth refute this view. It should be remembered that the enamel structure of a tooth is derived from the epiblast; that the dental pulp is derived from the mesoblast; that the deposition of dentin begins without and proceeds inwardly; that the deposition of the cementum on the roots of teeth begins within and proceeds outwardly, and that the blood supply to the peridental membrane is separate and distinct from the blood of the pulp.

"Perfect histological preparations show that the cementoblast receives its nourishment through the vascular supply of the peridental membrane, and recently stained and prepared specimens by Dr. Box, of Toronto, show conclusively that the nutrition of the cementoblast is derived from the perivascular tissues of the teeth. Therefore the tooth does not become a foreign body when its pulp has been removed. I have at the present time recently prepared specimens which confirm this view. Therefore, I fail to see the logic of extracting devitalized teeth when their investing structures evidence no gross pathology and the individual is in good health."—Dr. Ottolengui, in *Dental Items of Interest.*

---

STRESS AND STRAIN IN BRIDGE WORK. There is yet a paper to be written which fully and graphically notates the wonders of those true mechanical principles which are involved in correct occlusion and articulation, showing how natural teeth, perfectly aligned, minimize to the least possible degree undue stress in this process. Show me a bridge in which these principles have been correctly followed out!—A. E. Schneider, in *Items* for March.

---

STIFFENING CANAL POINTS. Gutta-percha canal points may be stiffened to any degree desired, and at the same time rendered sterile, by using tincture of iodine in glass point container. Flow iodine over points just enough to color them, but not to cover them. Allow to evaporate. Point in twenty-four hours will be ready for use. If not stiff enough to suit, repeat until point is dark brown. Iodine crystals seem to vulcanize the gutta-percha and form a coating, rendering the points sterile under almost all conditions.—*Digest.*

ANOTHER CASE OF FOCAL INFECTION. After serving two years in the army I was returning from New York to Cincinnati to resume my college course, and it so happened that a lady of about thirty years of age and wearing dark glasses with one eye encased in gauze occupied the Pullman seat opposite me. It was quite natural that we should find occasion for conversation, and I soon found that the lady was quite talented and interesting. She had been finishing her musical education at New York City and related to me the troubles she had encountered which forced her to give up her course. She was then on her way to her home in Kentucky to perhaps spend the rest of her days in total blindness.

It was during the winter of 1918 and spring of 1919 that she first had an attack of several severe head colds, all the bones of the face and head aching, and the loss of the senses of smell and taste. After the last cold in May, when her head was bent forward, a stream of clear water exuded from the left nostril. She immediately visited a nose and throat specialist in New York City during the month of June and was informed that she had "rose fever."

About the first of July, while on her way from Glen Ridge to New York, she thought a cinder was blown into her left eye. At the same time there appeared a big black speck before the eye which soon spread out into a comet shape. The cinder annoyed her greatly, yet could not be located. The eye gradually filled with black specks, which looked like drops of water under a microscope. She again went to see one of the leading eye and nose specialists of New York City, who made two thorough examinations of the eye, the second dilating the pupil, but he could find nothing wrong. He could not see the specks and informed the patient that they would probably disappear.

Three weeks later (August 22nd) a black cloud suddenly obscured the sight of the left eye, completely blinding it. The patient at this time again visited the first specialist, who made an examination of the eyes, the ears, the heart and the lungs, trying to discover the cause of her trouble, but found none. A blood test was then made, and an X-ray picture of the head taken, the result showing an infection of the sinuses. Here she was again sent to another nose and throat specialist in New York City. After his examination he informed her that the sinuses were the seat of all the trouble, and tried to wash out the antrum through the nose. This forced the infection farther in, making it a good deal worse. Here again she was directed to another nose specialist and he treated the sinuses by establishing drainage and using adrenalin and argyrol. This treatment was followed for a couple of weeks with a slight improvement in her vision (1/200). She was now sent to the Mountain Side Hospital, Montclair, N. J., for rest and further treatment. She learned at this time that the retina of the eye was unharmed and she decided to return to her home in Kentucky.

Of course, after listening to her story and noting that all the examinations were made without consideration of the teeth, and knowing that focal infections of the upper teeth, especially of the bicuspids or teeth in the region of the antrum, could be the cause of the troubles of the eye, I advised her upon reaching home to at once visit her dentist and have him X-ray all her teeth. I later heard from her and she informed me that the X-ray pictures showed two teeth abscessed, one in the lower jaw, a right lateral incisor with a granuboma at the apex, the other an upper second bicuspid with pus discharging into the antrum, and with a considerable portion of one of the roots necrosed. Upon physical examination these teeth appeared to be quite healthy. A few of the teeth contained fillings, but the teeth in question had never been treated.

The teeth were extracted and their sockets curretted, and an opening was made into the antrum for drainage. The ethmoid, spenoid, frontal and maxillary sinuses on the left side were all infected and about a teacupful of pus was removed during the treatment. The treatment was carried on through the nose and the opening was made in the upper bicuspid region for ten weeks, the tonsils and adenoids being later removed. The patient has fully recovered and is enjoying today a vision of twenty-twenty (20-20).—Arthur L. Roseberg, in *Cosmos* for June, 1920.

---

WHAT IS DONE TO EDUCATE THE PUBLIC BY THE MEDICAL PROFESSION. "Is there any good reason why the medical profession as a whole should not tell the people of the country as a whole how tuberculosis develops and how it can be avoided by proper food and plenty of fresh air?" No. And, as a matter of fact, the Council of Health and Public Instruction has for years been doing this very thing. In co-operation with the National Tuberculosis Association, the American Medical Association has printed hundreds of thousands of pamphlets written in every-day English and dealing with the very points suggested by him. That other great scourge, cancer, has also been dealt with in a series of inexpensive pamphlets. Of still another series on the conservation of vision, comprising twenty pamphlets and dealing with such subjects as "School Children's Eyes," "Wearing Glasses," "Eye Strain," "Care of the Eyes," etc., nearly two and one-half million have gone to the public. Nor has the care of the baby been neglected. The "Baby Welfare Pamphlets" give directions for mothers, telling them what to do before the baby comes and how to care for it after it arrives. Over a million of these have been put into the hands of those directly interested. A standard score card for public health conferences also has been prepared and more than half a million of these have gone out. In addition to these pamphlets, the Council on Health and Public Instruction has prepared educational posters and cartoons on various phases of public health questions and they are so designed as to appeal to the public.

To abolish as patients those who read themselves "sick" from the lurid symptom lists of the "patent medicine" advertisements, the *Journal of the American Medical Association* has, through its Propaganda for Reform Department, been giving the public the facts relative to the "patent medicine" evil and quackery for over a decade. Over a million pamphlets and books dealing with the nostrum evil and quackery have been put in the hands of the public and the association has spent hundreds of thousands of dollars in maintaining the organization necessary to prepare this educational work.

Thousands of individual letters from laymen and physicians on the subject are answered yearly and without cost; educational placards on the same subject have been prepared and are being used by various organizations interested in the public health, not only throughout the United States, but in various parts of the world; illustrated public lectures have been given on the same subject; lantern slides have been made so that stereopticon lectures can be delivered; information is furnished free to advertising clubs, newspapers and magazines; large business organizations which look after the health and safety of their employees call on the propaganda department for the material it has for educational purposes.—*Journal N. D. Assn.*

---

Dental Health. Our statistics upon disease are very incomplete. Even a national census can report only a few facts in regard to the general population. It is customary for those who have physical imperfections to conceal them whether the imperfection is the result of birth, accident or disease.

Since the public school dental examinations have been carried on, the relative figures have been surprising and we are convinced that more than ninety per cent. of all children of school age are suffering from some form of decay of the teeth or other derangement of the mouth.

The American Public Health Association states that, of the 110,000,000 citizens of this country, 45,000,000 are physically imperfect; 1,500,000 die every year; 3,000,000 never leave their beds; 1,000,000 have tuberculosis; and from 2,000,000 to 3,000,000 have hookworm and malaria. Only 37,500,000 are fairly healthy and 19,500,000 in full vigor.

We are not even positive that less than one-fifth of our population are in full vigor because there may be among those people much that is concealed and many incipient diseases that are as yet not sufficiently developed to be recognized.

The matter of health of every portion of the body is far more vital than any other one thing at present.

The greatest cause of disease is ignorance of well-established facts. Even those who have given the closest attention to health and hygiene realize very definitely that we have only scaled the surface of the possibilities of life and the few things we actually do know well are not widely understood. In teaching the principles of hygiene as we know them, we must not get the idea that all that will ever be known is known and that this is the final advance.

We are simply beginning to recruit the volunteers who will carry the message as we know it to those with whom we do not come in contact.

In the old civilizations, such as that of ancient Greece, the Spartans regarded childhood from the standpoint of physical perfection. If the child lacked in vitality and was afflicted with any weakness, it was cast aside to die and in many cases actually put to death. Until the last few years, we were not so far better than this, because comparatively little attention was paid to child welfare and the insidious infections were allowed to do *slowly* the work that the old Spartan did *quickly*.

One of the greatest contributory causes of disease, under-development, under-nourishment, and in many instances, of mental deficiency, in children, is an unhealthy mouth.

The highest death rate among children is from two to six years of age—the pre-school period. During this period, the child depends wholly upon its temporary teeth to prepare its food for the action of the digestive ferments. This is a time of very rapid growth when the cells of the little body are constantly calling for nourishment—not only to maintain life, but to increase the size and activity of the child. If, at this time, we allow the important organs of mastication to become deficient, to that extent we lower the vitality of the child because the food—even though sufficient in quality and quantity—will not be properly prepared for the chemical action of the stomach and intestines and many of the elements, we believe, frequently the vitamines themselves, may be lost so far as benefit to the child is concerned—because of this improper mastication of food.

The effort of teaching mouth hygiene to school children must begin in the pre-school period to be a success. The earlier we can reach parents and guardians and convince them that temporary teeth are just as important—in fact, more important—to the growing child than the permanent teeth themselves are to the adult, the better.—Extracted from a paper by Dr. Rea Proctor, in *Oral Hygiene* for June.

---

The First Dental School-Clinic. Dr. Jessen, of Basle, Switzerland, makes a clear and accurate statement that seems to show conclusively that he established the first school-clinic for dental hygiene in Strassburg, Alsace, in 1888. This antedates the clinic established by Dr. George Cunningham in Cambridge, England.

Articulation of Artificial Teeth. At the present time, as well as in the time since Bonwill's methods were introduced, there seems to be considerable unrest in regard to adjusting artificial dentures, and perhaps it may not be out of place to present just one more, which has proved very satisfactory for many years.

When one remembers that in mastication the power exerted ranges from almost nothing to over two hundred pounds, but averaging, perhaps, from twelve to twenty odd; and that the artificial dentures are only held in place by their adaptation (not being by any means fixtures), and are not always easy to hold in place, even under the most favorable circumstances, it seems well to adapt conditions to circumstances. By making the dentures so that the direction of the force of mastication shall be as much as possible to the center of the arch, and making the contact points under all circumstances small, the results obtained should be that of most efficiency.

By placing the teeth so that they rest, as much as possible, directly over the alveolar ridge, avoiding *any* contact of the six anterior teeth, allowing only the bicuspids and first molars to touch, and shaping these so that they only touch on the palatal and lingual edges, and to the extent that there will be a slight triangular space between the teeth with the base of the triangle pointing externally, there results a condition by which, when the jaws are at rest, the dentures are kept in place, and while masticating the greatest force on the smallest contact points may be exerted and with the tendency always to steady both dentures. Naturally, the shorter the teeth the greater the stability.—Dr. C. Keyes, Rio de Janeiro, in *Cosmos* for June, 1920.

